# High‐Performance Isotropic Thermo‐Electrochemical Cells Using Agar‐Gelled Ferricyanide/Ferrocyanide/Guanidinium

**DOI:** 10.1002/gch2.202200207

**Published:** 2023-04-07

**Authors:** Lixian Jiang, Shohei Horike, Masakazu Mukaida, Kazuhiro Kirihara, Kazuhiko Seki, Qingshuo Wei

**Affiliations:** ^1^ Nanomaterials Research Institute Department of Materials and Chemistry National Institute of Advanced Industrial Science and Technology (AIST) 1‐1‐1 Higashi Tsukuba Ibaraki 305‐8565 Japan; ^2^ Department of Chemical Science and Engineering Graduate School of Engineering Kobe University 1‐1 Rokkodai‐cho Kobe 657‐8501 Japan; ^3^ PRESTO Japan Science and Technology Agency Kawaguchi 332‐0012 Japan; ^4^ Research Center for Membrane and Film Technology Kobe University 1‐1 Rokkodai‐cho Kobe 657‐8501 Japan; ^5^ GZR National Institute of Advanced Industrial Science and Technology (AIST) 16‐1 Onogawa Tsukuba Ibaraki 305‐8569 Japan

**Keywords:** agar, gelled electrolytes, guanidinium, isotropic, thermo‐electrochemical cells

## Abstract

An isotropic thermo‐electrochemical cell is introduced with a high Seebeck coefficient (*S*_e_) of 3.3 mV K^−1^ that uses a ferricyanide/ferrocyanide/guanidinium‐based agar‐gelated electrolyte. A power density of about 20 µW cm^−2^ is achieved at a temperature difference of about 10 K, regardless of whether the heat source is on the top or bottom section of the cell. This behavior is very different from that of cells with liquid electrolytes, which exhibit high anisotropy, and for which high *S*_e_ values are achieved only by heating the bottom electrode. The guanidinium‐containing gelatinized cell does not exhibit steady‐state operation, but its performance recovers when disconnected from the external load, suggesting that the observed power drop under load conditions is not due to device degeneration. The large *S*_e_ value and isotropic properties can mean that the novel system represents a major advancement from the standpoint of harvesting of low‐temperature heat, such as body heat and solar thermal heat.

## Graphical abstract

Here an isotropic thermo‐electrochemical cell is presented using a ferricyanide/ferrocyanide/guanidinium‐based agar‐gelated electrolyte. A power density of ≈20 µW cm^−2^ is achieved at a temperature difference of ≈10 K, regardless of whether the heat source is at the top or bottom. The isotropic properties suggest that the system represents a major advance in terms of low‐temperature heat harvesting.

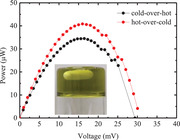

## 1 Introduction

In the face of the global issues of energy supply, global warming, and decarbonization, harvesting low‐grade waste heat (<200 °C) is an urgent goal and hot research topic.^[^ ^1^, ^2^ ^]^ Several thermal energy conversion phenomena, such as the Seebeck effect,^[^ ^3^ ^]^ the thermogalvanic effect,^[^ ^4^, ^5^, ^6^ ^]^ and the Soret effect,^[^ ^7^, ^8^, ^9^ ^]^ have been explored.

Thermoelectric devices based on the Seebeck effect convert heat directly into electricity and are promising candidates to realize the harvest of heat energy. The most common low‐temperature thermoelectric materials are based on semiconducting materials characterized by a Seebeck coefficient (*S*_e_) of ≈100 µV K^−1^, like Bi_2_Te_3_.^[^ ^10^ ^]^ Notably, Te is an expensive, rare metal, and its use is not environmentally friendly, making this chemical species unsuitable for large‐scale applications.

Thermo‐electrochemical cells (also called thermogalvanic cells or thermocells) are based on redox reactions, like the ferrocyanide/ferricyanide [Fe(CN)_6_^4−^/Fe(CN)_6_^3−^] reaction.^[^ ^11^, ^12^, ^13^, ^14^ ^]^ Imposing a temperature difference between two electrodes will generate a large Seebeck coefficient of a few millivolts per degree kelvin.^[^ ^15^, ^16^, ^17^, ^18^, ^19^, ^20^, ^21^ ^]^ This phenomenon descends from the fact that the size of ions is much larger than that of electrons, which shows significant reaction entropy.^[^ ^6^, ^22^, ^23^ ^]^ Very recently, for thermo‐electrochemical cells, an *S*_e_ value as high as ≈3.7 mV K^−1^ was reported on the basis of an aqueous system comprising Fe(CN)_6_^4−^/Fe(CN)_6_^3−^ and guanidinium cations (Gdm^+^).^[^ ^24^, ^25^ ^]^ Such a high value for the *S*_e_ is attributed to the configuration entropy change associated with the selective binding of Gdm^+^ to Fe(CN)_6_^4−^.^[^ ^26^, ^27^ ^]^ On the other hand, with the addition of Gdm^+^, the high *S*_e_ value could only be achieved as the cells are heated from the bottom. This observation descends from the fact that the selective binding of Gdm^+^ to Fe(CN)_6_^4−^ results in the formation at room temperature of a high density crystalline precipitate near the bottom of the thermocell.^[^ ^24^ ^]^ Since the said crystalline precipitate in the solution exhibits a strong temperature‐dependent solubility, the formation at high temperatures of a solid‐state electrolyte with a homogeneous matrix may be a possible route to solving the cell anisotropicity issue. Solid‐state electrolytes have been fabricated using various gelling agents,^[^ ^28^, ^29^ ^]^ like cellulose,^[^ ^30^ ^]^ water‐soluble polymers,^[^ ^31^, ^32^ ^]^ and superabsorbent polymers.^[^ ^33^ ^]^ Recently, Zhou et al. reported a porous material as a matrix for thermo‐electrochemical applications.^[^ ^34^ ^]^ Interestingly, this system seems universal and shows fast diffusion of the electrolyte. On the other hand, the introduction of guanidine hydrochloride (GdmCl) into the polymer matrix does not improve the *S*_e_ of Fe(CN)_6_^4−^/Fe(CN)_6_^3−^ much, which may be due to the difficulty in controlling the dispersion of crystalline precipitates. Considering the fabrication procedures, the gelation temperature, and the operating temperature of thermo‐electrochemcial cells based on ferrocyanide/ferricyanide/guanidinium, agar could be a straightforward choice as a gelling agent.^[^ ^35^ ^]^ We can heat the mixture to its melting point (>70 °C) and leave it to cool down to room temperature to form a gel electrolyte. Notably, use of a gel electrolyte instead of a liquid electrolyte also maximizes temperature differences by eliminating convection. This consideration is important for low‐temperature energy harvesting, such as that involved in wearable devices, which utilize body heat.

In this study, we report a solid‐state redox electrolyte formed by agar and a Fe(CN)_6_^4−^/Fe(CN)_6_^3−^/Gdm^+^ solution. The solid‐state electrolyte exhibits an *S*_e_ value of 3.3 mV K^−1^, almost identical to that of the liquid electrolyte. Notably, the solid‐state thermocell based on the said electrolyte displays an isotropic nature, which is an advantageous feature with respect to liquid electrolytes. For this thermocell, a power density of ≈20 µW cm^−2^ was achieved at a temperature difference of ≈10 K, regardless of whether the heat source was on the cell's top or bottom. We also carried out a continuous power generation experiment using an external resistor. Similarly to liquid cells, the gelled cells containing Gdm^+^ do not show the steady‐state operation, but their performance recovers once the device is disconnected from the external loading. The total energy density of the gelled cells during 1 h of loading is close to that reported for the best liquid cells. The results of the present work suggest that agar‐gelled electrolytes could be ideal electrolyte candidates for the manufacture of solid‐state thermo‐electrochemical cells.

## 2 Results and Discussion

**Figure 1:**
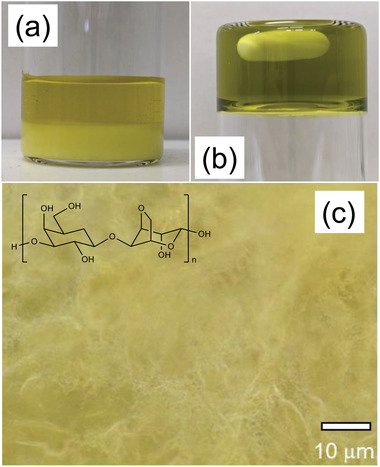
Photographs of a) the ferricyanide/ferrocyanide/guanidinium electrolyte solution and b) gel prepared using 1 wt% agar with the ferricyanide/ferrocyanide/guanidinium solution. c) Microscopic image of the gel after freeze‐drying. In the inset, is reported the chemical structure of agar.

Photographs of the 0.4 m potassium ferricyanide (K_3_[Fe(CN)_6_])/0.4 m potassium ferrocyanide (K_4_[Fe(CN)_6_])/1 m GdmCl solution and of the relevant gel formed using agar in screw‐cap vials are reported in **Figure** **1**a,b, respectively. A crystalline precipitate can be observed in Figure 1a, which was produced by the selective binding of Gdm^+^ to Fe(CN)_6_^4−^. Agar (1 g) was added to 100 mL of solution, and the obtained mixture was heated to 70 °C under stirring. At this temperature, the crystalline precipitate was observed to fully dissolve in the solution, and the gel was observed to form during the cooling process. The gel electrolyte kept a transparent state, and the results of the inversion test confirmed gel formation, as can be evinced from Figure 1b. Figure 1c is a microscopic image of the gel after undergoing freeze‐drying. This image demonstrates that the structure of the gel is that of a network of macropores of several micrometers in diameter. A similar approach could be used to form the gel without adding GdmCl, as shown in Figure S1 (Supporting Information).

**Figure 2:**
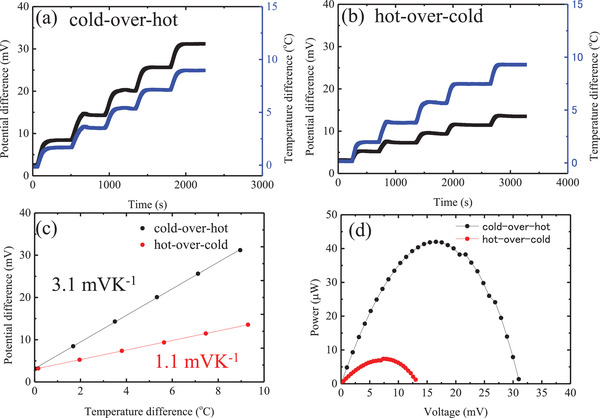
Temporary change in potential difference measured using Pt electrodes with the imposed temperature difference between the electrodes for a) a solution of ferricyanide/ferrocyanide/guanidinium electrolyte, where the temperature of the top electrode (cold) was controlled at 25 °C (cold‐over‐hot electrode arrangement), and b) a solution of ferricyanide/ferrocyanide/guanidinium electrolyte, where the temperature of the bottom electrode (cold) was controlled at 25 °C (hot‐over‐cold electrode arrangement). c) Plot of the open‐circuit voltage as a function of temperature difference between electrodes for a solution of ferricyanide/ferrocyanide/guanidinium with cold‐over‐hot (black dot) and hot‐over‐cold (red dot) electrode arrangements. d) Power output of the device using solution of ferricyanide/ferrocyanide/guanidinium with cold‐over‐hot (black dot) and hot‐over‐cold (red dot) electrode arrangements, when the temperature of the hot side was 35 °C and that of the cold side was 25 °C.

The *S*_e_ was measured using the homemade setup described in the “Experimental Section” and in Figure S2 (Supporting Information). Fe(CN)_6_^4−^ with low solvation entropy is spontaneously oxidized to Fe(CN)_6_^3−^ with high solvation entropy by releasing electrons to the high‐temperature electrode, and therefore, we have fixed *S*_e_ to a plus sign here. In our experience, the Seebeck coefficient measured with this setup may contain an error of about 10%. One reason for this is that temperature and voltage are not measured at exactly the same point. Another reason is that offside sometimes occurs at the start of the measurement. We found that the cell voltage did not reach zero despite the absence of temperature differences. This is thought to be due to the fact that ion diffusion at the gel electrolyte–electrode interface is not as uniform as at the solution electrolyte–electrode interface, resulting in the generation of offsides. Before measuring the temperature dependence of voltage, the electrodes were short‐circuited to suppress the occurrence of offsides. In **Figure** **2**a,b, are reported data reflecting the voltage change in open‐circuit conditions measured under the indicated imposed temperature difference using Pt electrodes for 0.4 m K_3_[Fe(CN)_6_]/0.4 m K_4_[Fe(CN)_6_]/1 m GdmCl solution for the cold‐over‐hot and hot‐over‐cold electrode configurations, respectively. The surface area of the cell was 1.8 cm^2^, and the distance between the cold and hot electrodes was 1.0 cm. The cold electrode temperature was fixed at a value of 25 °C, and that of the hot electrode was gradually made to increase from 25 to 35 °C at incremental steps of 2 °C. The voltage was found to increase proportionally to the temperature difference. The voltage also exhibited a stable response at a constant temperature difference in both configurations. The *S*_e_ is extracted from the voltage as a function of temperature difference at a steady state. As can be evinced from Figure 2c, the cell with a solution of 0.4 m K_3_[Fe(CN)_6_]/0.4 m K_4_[Fe(CN)_6_]/1 m GdmCl was characterized by a value for the slope of 3.1 mV K^−1^ in the cold‐over‐hot configuration and one of 1.1 mV K^−1^ in the hot‐over‐cold configuration, which indicates that enhanced *S*_e_ values can only be observed when the bottom electrode is heated, suggesting that the liquid electrolyte is characterized by high anisotropy. This characteristic descends from Fe(CN)_6_^4−^ ions concentrating in the bottom electrode due to the high density of the crystal. Indeed, heating the top electrode could not effectively push the oxidation reaction due to the low concentration of Fe(CN)_6_^4−^ ions.

The maximum power output of the devices could be determined by conducting a current–voltage scan from the open‐circuit voltage to the short‐circuit condition (Figure 2d; Figure S3, Supporting Information). In the cold‐over‐hot electrode configuration, the liquid cell based on the 0.4 m K_3_[Fe(CN)_6_]/0.4 m K_4_[Fe(CN)_6_]/1 m GdmCl solution exhibited a power output of more than 40 µW (corresponding to a power density of 23 µW cm^−2^), which is more than five times as high as the power output of the same device with a hot‐over‐cold electrode configuration (power density = 4 µW cm^−2^).

**Figure 3:**
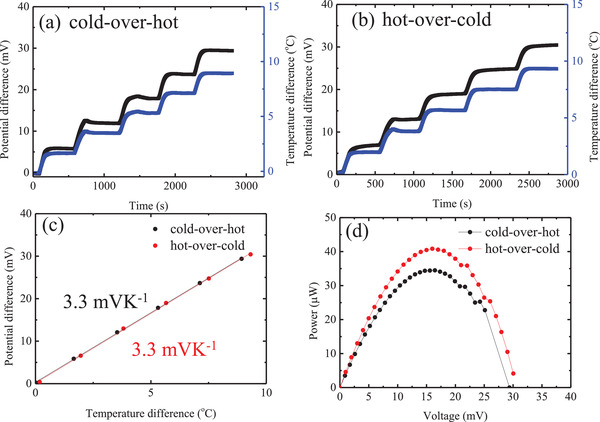
Temporary change in potential difference measured by Pt electrodes with the imposed temperature difference between the electrodes for a) gel of the ferricyanide/ferrocyanide/guanidinium electrolyte, where the temperature of the top electrode (cold) was controlled at 25 °C (cold‐over‐hot electrode arrangement), and b) gel of the ferricyanide/ferrocyanide/guanidinium electrolyte, where the temperature of the bottom electrode (cold) was controlled at 25 °C (hot‐over‐cold electrode arrangement). c) Plot of the open‐circuit voltage as a function of the electrode temperature difference for gels of ferricyanide/ferrocyanide/guanidinium with cold‐over‐hot (black dot) and hot‐over‐cold (red dot) electrode arrangements. d) Power outputs of the devices using gels of ferricyanide/ferrocyanide/guanidinium characterized by a cold‐over‐hot (black dot) and of hot‐over‐cold (red dot) electrode arrangements, when the temperature of the hot side was 35 °C and that of the cold side was 25 °C.

In **Figure** **3**a,b, are reported open‐circuit voltage values under applied temperature difference in the agar‐gelled electrolyte (0.4 m K_3_[Fe(CN)_6_]/0.4 m K_4_[Fe(CN)_6_]/1 m GdmCl) measured using a Pt electrode. The size of the cell and the distance between the electrodes are identical to the corresponding parameters described for the liquid cells. As can be evinced from Figure 3a,b, the voltage increased proportionally to the temperature difference, a similar relationship to the one inferred from the data in Figure 2. At a steady state, we extracted from the data in Figure 3a,b the value of *S*_e_. As shown in Figure 3c, the cell with gel electrolyte exhibited a slope of 3.3 mV K^−1^ in both electrode configurations (i.e., cold‐over‐hot and hot‐over‐cold configurations). This value is much larger than the one obtained for the agar‐gelled electrolyte lacking GdmCl (and containing 0.4 m K_3_[Fe(CN)_6_]/0.4 m [K_4_Fe(CN)_6_)]), for which an *S*_e_ value of 1.7 mV K^−1^ was determined (Figure S4, Supporting Information). The mentioned difference in the *S*_e_ value could be attributed to the configuration entropy change associated with the selective binding of Gdm^+^ to Fe(CN)_6_^4−^. Notably, in the agar‐gelled electrolyte, the Fe(CN)_6_^4−^, Fe(CN)_6_^3−^, and Gdm^+^ ions are homogeneously distributed in the polymer matrix, which could be different from gel electrolytes using the porous matrix.^[^ ^34^ ^]^ Therefore, a high value for *S*_e_ was observed whether the heat source was placed in correspondence of the bottom electrode or the top electrode. The large *S*_e_ and isotropic properties of the described system could constitute a major advancement in the utilization of low‐temperature heat, such as body heat and solar thermal heat.^[^ ^36^ ^]^

Based on the results of a current–voltage scan (Figure 3d), the device exhibited a power output of more than 40 µW (corresponding to a power density of 23 µW cm^−2^) with a hot‐over‐cold electrode configuration, which was slightly higher than the corresponding parameter for the device with a cold‐over‐hot electrode configuration (power density = 19 µW cm^−2^). Please note that the *S*_e_ is equivalent regardless of cold‐over‐hot or hot‐over‐cold configuration. This difference has to do with the fact that a more significant temperature difference is achieved in a hot‐over‐cold electrode configuration, due to the density gradient in the flow field (differences in convective heat transfer). A closer look at Figure 3c shows that the temperature difference is always larger in the hot‐over‐cold electrode configuration, even if the temperature setting is the same. The power output is close to that of the liquid cell in the cold‐over‐hot electrode arrangement reported in Figure 2. Although this result seems curious, it actually makes sense, and the results of impedance measurements suggest that the ionic conductivity in the gelled electrolyte is not significantly reduced with respect to the corresponding liquid electrolyte (Figure S5, Supporting Information). This observation may be explained by the fact that the weight ratio of agar to water is 1:100 and the ionic migration is not very affected at high frequencies.

We could estimate the efficiency of the cells by calculating the heat flux *q* using Fourier's law^[^ ^37^ ^]^

(1) $$\begin{array}{c} q\;=\frac{\Delta T\kappa A}{d}\; \end{array}$$

where Δ*T* is the temperature difference, *κ* is the thermal conductivity of the electrolyte, *A* is the device area, and *d* is the distance between two electrodes. Using Δ*T* = 10 K, *A* = 1.8 cm^2^, *κ* = 0.5 W m^−1^ K^−1^, and *d* = 1 cm, the calculated heat flux is ≈90 mW. The maximum power output of the cells is 40 *µ*W, and therefore the efficiency *η* is ≈0.04%. The fraction of the theoretically limiting Carnot cycle efficiency, $\eta_{r}=\;\eta \;\frac{T_{h}}{\Delta T}\;≅1.2\%$, where *T*_h_ is the temperature at hot side. Note that the efficiency calculated at the maximum output could be overestimated because the device did not show steady‐state operation (see the latter part).

**Figure 4:**
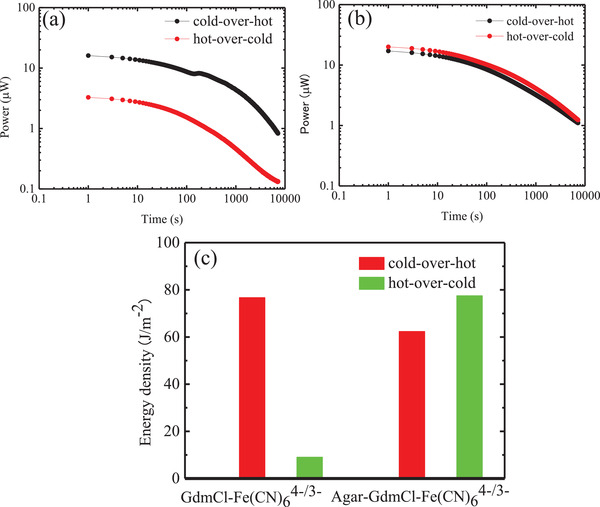
Plots of the power output of cells comprising a) ferricyanide/ferrocyanide/guanidinium electrolyte solution with external loading (40 Ω) or b) gel of the ferricyanide/ferrocyanide/guanidinium electrolyte as a function of the working time, whereby the cold electrode was above the hot electrode (cold‐over‐hot electrode arrangement) or vice versa (hot‐over‐cold electrode arrangement). c) Plot of the energy density recorded for a solution of ferricyanide/ferrocyanide/guanidinium electrolyte and for a gel of ferricyanide/ferrocyanide/guanidinium electrolyte, where the energy was calculated by integrating the power function with respect to time (1 h) reported in panels (a) and (b).

*I*–*V* scanning may be the easiest way to evaluate thermo‐electrochemical cells, especially those whose output is not stable. However, it is also important to know that depending on the scan time and cell geometry, it is possible to overestimate the results, as in a recent study by Aldous’ group.^[^ ^38^ ^]^ Therefore, they have also performed long‐time loading experiments and attempted to compare average power densities. We carried out the continuous power generation measurement with an external resistor of 40 Ω for 1 h (note that 40 Ω is greater than the internal resistance of the cell, so the power output will not be at its maximum). As can be evinced from the data reported in **Figure** **4**a, the initial power output of the device based on the ferricyanide/ferrocyanide/guanidinium solution was ≈20 µW, and decreased during working time in both electrode arrangements; this observation was different from that of the device based on the ferricyanide/ferrocyanide solution, which could quickly reach the stable state when connected to the external resistance of 40 Ω (Figure S6, Supporting Information). As the results of our previous studies indicated, these observations may have to do with the fact that the circulation of Fe(CN)_6_^3−^and Fe(CN)_6_^4−^in the cells is hampered by the difficulty of diffusion of Fe(CN)_6_^3−^in the crystal layer of (Gdm^+^)*_n_* [Fe(CN)_6_^4−^] and the slow precipitation of (Gdm^+^)*_n_* [Fe(CN)_6_^4−^] from the top electrode. As can be evinced from Figure 4b, the device's power output based on the gel of ferricyanide/ferrocyanide/guanidinium shows a closed tendency, which decreased during working time. Note that the output values for the hot‐over‐cold and cold‐over‐hot electrode configurations nearly overlap with each other with respect to time, suggesting that this system is isotropic. Although this trend is similar to that of liquid cells, the underlying mechanism may be different. In the gelled electrolyte, the ions are thought to be homogeneously distributed in the agar matrix, but there is no crystalline layer inside the gel. The decreased performance of the device containing the gelled electrolyte during loading may be related to the difficulty of the ions to pass through the porous structure. This hypothesis is supported by the fact that in loading experiments conducted using agar‐gelated Fe(CN)_6_^3−^/Fe(CN)_6_^4−^electrolytes, the cell performance decreased with time during loading. On the other hand, the liquid cell containing the Fe(CN)_6_^3−^/Fe(CN)_6_^4−^ solution exhibited stable operation under the same conditions (Figure S6, Supporting Information). If we normalize the starting point and compare decay rates, as shown in Figure S7 (Supporting Information), it was found that the gelled electrolyte decayed slightly faster than the aqueous electrolyte. The average power output in 100 s decreases to ≈10 µW.

As a way to estimate the length that the ions cover as they migrate, we calculate the travel distance at the loading time. For an output power of 20 µW and a 40 Ω load, as can be evinced from Figure 3b, the current value (*I*) is determined by Equation (2)

(2) $$\begin{array}{c} I\;=\sqrt{\frac{P}{R}}\;=\sqrt{\frac{20\;\times \;10^{-6}}{40}}\;A\;=\;7\times 10^{-4}\;A \end{array}$$

where *P* is the power output, and *R* is the loading resistance.

The charge transferred per second can be estimated via Equation (3)

(3) $$\begin{array}{ccc} \frac{I}{eN_{A}} & = & \frac{7\times 10^{-4}\;A}{1.6\times 10^{-19}\;C\times 6.02\times 10^{23}\;{\mathrm{mol}}^{-1}}\\ & = & 7.3\times 10^{-9}\;{\mathrm{mol}\;s}^{-1} \end{array}$$

where *e* is the elementary charge and *N*_A_ is Avogadro's constant. When the Fe(CN)_6_^3−^/Fe(CN)_6_^4−^electrolyte concentration is 0.4 mol L^−1^, the cell surface and area is 1.8 cm^2^, the corresponding ion moving distance *d* in 1 s is given by Equation (4)

(4) $$\begin{array}{ccc} d\; & = & \frac{7.3\times 10^{-9}\;{\mathrm{mol}\;s}^{-1}}{0.4\;{\mathrm{mol}\;L}^{-1}\times 1.8\;{\mathrm{cm}}^{2}}\\ & = & 1.0\times 10^{-5}\;{\mathrm{cm}\;s}^{-1}=100\;{\mathrm{nm}\;s}^{-1} \end{array}$$

Therefore, the ions moved by about 1 µm at 10 s loading and by about 10 µm at 100 s loading. As can be evinced from Figure 3, cell performance clearly began to decline after 10 s, and the range of decline became larger after 100 s. These observations suggest that the migrating ions may be facing a barrier of 1–10 µm in size, which is approximately the same as the size of the pores of the agar gel.

The corresponding energy densities in 1 h of cells with kinds of electrolytes are reported in Figure 4c. In the cold‐over‐hot electrode arrangement, the energy density of the cell comprising the gel of ferricyanide/ferrocyanide/guanidinium has a value of 78 J m^−2^, almost identical to that of the cell comprising the solution of ferricyanide/ferrocyanide/guanidinium (77 J m^−2^). In the hot‐over‐cold electrode configuration, the cell comprising the solution of ferricyanide/ferrocyanide/guanidinium is characterized by an energy density of only 8 J m^−2^. By contrast, the energy density of the cell comprising the gel of ferricyanide/ferrocyanide/guanidinium in a cold‐over‐hot electrode arrangement is 62 J m^−2^.

**Figure 5:**
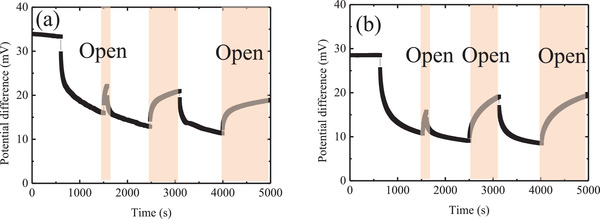
Temporary change in voltage during cell connection and disconnection from the external loading (40 Ω) for devices based on a) the solution of ferricyanide/ferrocyanide/guanidinium and b) the gel of ferricyanide/ferrocyanide/guanidinium. In both cases, the cold electrode was placed above the hot electrode.

We further monitored the voltage change by reversibly connecting and disconnecting the external loading (40 Ω). In **Figure** **5**a,b, are reported data reflecting the temporary change of voltage observed for devices based on liquid and gel electrolytes when the relevant cells were connected to and disconnection from the external loading (40 Ω). The voltage of both the cells comprising the solution electrolyte and the gelled electrolyte decreased when the said cells were connected to the external loading; by contrast, the voltage slowly recovered when the cells were disconnected from the external loading. Notably, the voltage recovery seems to take some time, probably as a result of the slowness of ion diffusion. Nevertheless, we found that when we remove the heat source and return to room temperature for a few hours, the cell can return to its initial performance, including the exhibition of the original values for the *S*_e_ and internal resistance. This observation suggests that the decrease in voltage during loading is not due to the degeneration of the device but, most likely, to the slowness of the circulation of the ions inside the cells.

In the initial state, when no heat source has been applied, the density of ions in the gel electrolyte is homogeneous. Once the electrode is heated, however, the free Fe(CN)_6_^4−^ and Fe(CN)_6_^3−^ ions accumulate near the two electrodes to achieve an open‐circuit voltage. When the device is connected to an external resistor, Fe(CN)_6_^4−^ gives out electrons, forming Fe(CN)_6_^3−^ on the hot side, while Fe(CN)_6_^3−^ accepts electrons from the electrode at the cold side from Fe (CN)_6_^4−^. For continuous power generation, both the reduced species Fe(CN)_6_^4−^ and the oxidized species Fe(CN)_6_^3−^ need to diffuse through a porous network structure to reach the counter electrodes. Ion circulation may not be efficient due to the blocking effect of the agar fiber mesh, which leads to the gel‐based device not showing a steady‐state operation. Nevertheless, the performance recovers if we wait for a long enough period of time. Since the total energy density of the gelled cell was close to that of the best liquid cell, evidence suggests that the agar‐gelled electrolyte could be an ideal candidate for the manufacture of a solid‐state thermoelectric conversion cell. However, achieving the steady‐state operation remains a challenge when this cell is used.

## 3 Conclusion

In this study, were developed isotropic thermo‐electrochemical cells with a high *S*_e_ value of 3.3 mV K^−1^ using agar‐gelled electrolytes based on ferricyanide/ferrocyanide/guanidinium. A power density of ≈20 µW cm^−2^ was achieved at a temperature difference of ≈10 K, regardless of whether the heat was supplied at the cells’ top or bottom. This behavior is significantly different from that of cells using liquid electrolytes, which exhibit high anisotropicity. In the agar‐gelled electrolyte, the Fe(CN)_6_^4−^, Fe(CN)_6_^3−^, and Gdm^+^ ions are homogeneously distributed in the polymer matrix, because the said matrix forms from a solution at high temperatures (≈70 °C). In fact, at these temperatures, the crystalline precipitate originally present at the bottom of the solution container is fully dissolved. The high *S*_e_ value could be attributed to the configuration entropy change associated with the binding of Gdm^+^ to Fe(CN)_6_^4−^. Remarkably, however, the devices constructed with agar‐gelled electrolytes do not display a steady‐state operation, probably due to an inefficient ion circulation in the agar matrix. Nevertheless, the said devices’ performance recovered when they were disconnected from the external loading and the heat source was removed, suggesting that the decrease in voltage during loading is not due to the degeneration of the device. The systems developed herein are not perfect in terms of continuous power generation, but their high *S*_e_ value and isotropic nature could be a major step forward in harvesting low‐temperature heat, such as body heat. In addition, attempts are being made to improve ion circulation inside the described devices by controlling the size of the gel and by combining other forms of energy, such as vibrations, with heat.

## 4 Experimental Section

### Chemicals

The chemical reagents K_3_[Fe(CN)_6_], K_4_[Fe(CN)_6_], and agar powder were purchased from Fujifilm Wako Pure Chemical Corporation. GdmCl was purchased from Tokyo Chemical industry Co., Ltd. All solutions were prepared using deionized water.

### Fabrication of Gel Electrolytes

The aqueous solutions of ferricyanide/ferrocyanide (0.4 m K_3_[Fe(CN)_6_]/0.4 m K_4_[Fe(CN)_6_]) or ferricyanide/ferrocyanide/guanidinium (0.4 m K_3_[Fe(CN)_6_]/0.4 m K_4_[Fe(CN)_6_]/1 m guanidine hydrochloride) with agar were heated up to the boiling point; subsequently, the boiled solutions were placed in the cell and were allowed to cool down to room temperature to produce the gel electrolytes. The implemented ratio was 1 g of agar in 100 mL of solution; notably, the solution with agar was stirred as it was being heated.

### Characterization

The thermovoltage was determined using a homemade setup. The temperature at the two electrodes was controlled using two controllers (Ampere UTC‐200A) with Peltier modules. K‐type thermocouples were inserted into a small hole on the side of the electrode, a setup that can ensure the accuracy of the temperature measurement on the electrode. Temperature data on the two electrodes were collected, while the voltage difference between them was measured using a voltage/temperature unit (HIOKI U8500). The electrical characteristics of the cells were measured with a semiconductor parameter analyzer (KEITHLEY 2450). The current–voltage characteristics were measured using the linear sweep voltammetry method. The scans started from the open‐circuit voltage to 0. The scan rate was ≈5 mV s^−1^. The quadratic parabola of the relationship *P* = *IV* = *V* ^2^/*R* was extracted from the *I*–*V* curve. The morphology of the gel electrolyte was determined using a digital microscope (Keyence VHX‐7000).

The *S*_e_ of a thermo‐electrochemical cell with two Pt electrodes was evaluated using the homemade setup, whereby the distance between the two electrodes was 1.0 cm and the area was 1.8 cm^2^. The temperature difference between the electrodes was maintained between 0 and 10 °C, wherein the cold electrode was held at a temperature of 25 °C. All cells were measured in a thermally “vertical” orientation, wherein the hot electrode was above the cold electrode (hot‐over‐cold electrode arrangement) or the cold electrode was above the hot electrode (cold‐over‐hot electrode arrangement).

## Conflict of Interest

The authors declare no conflict of interest.

## Supporting information

Supporting Information

## Data Availability

The data that support the findings of this study are available from the corresponding author upon reasonable request.
