# Supplementary material for: High‐Performance Isotropic Thermo‐Electrochemical Cells Using Agar‐Gelled Ferricyanide/Ferrocyanide/Guanidinium
Source: Glob Chall. 2023 Apr 7;7(6):2200207. doi: 10.1002/gch2.202200207 (PMC10242534; doi:10.1002/gch2.202200207)
Supplement: Supplementary file 1 — Supporting Information [file GCH2-7-2200207-s001.pdf]

Global Challenges
Open Access

Supporting Information

for *Global Challenges.*, DOI 10.1002/gch2.202200207

High-Performance Isotropic Thermo-Electrochemical Cells Using Agar-Gelled Ferricyanide/Ferrocyanide/Guanidinium

*Lixian Jiang, Shohei Horike, Masakazu Mukaida, Kazuhiro Kirihara, Kazuhiko Seki and Qingshuo Wei**

Supporting Information

High-performance Isotropic Thermoelectrochemical Cells Using Agar-gelled Ferricyanide/Ferrocyanide/Guanidinium

Lixian Jiang, Shohei Horike, Masakazu Mukaida, Kazuhiro Kirihara, Kazuhiko Seki and Qingshuo Wei,*

L. Jiang, M. Mukaida, K. Kirihara, Q. Wei
Nanomaterials Research Institute, Department of Materials and Chemistry, National Institute of Advanced Industrial Science and Technology (AIST), 1-1-1 Higashi, Tsukuba, Ibaraki 305-8565 Japan

S. Horike
Department of Chemical Science and Engineering, Graduate School of Engineering, Kobe University, 1-1 Rokkodai-cho, Kobe 657-8501, Japan

PRESTO, Japan Science and Technology Agency, Kawaguchi 332-0012, Japan

Research Center for Membrane and Film Technology, Kobe University, 1-1 Rokkodai-cho, Kobe 657-8501, Japan

K. Seki
GZR, National Institute of Advanced Industrial Science and Technology (AIST), 16-1 Onogawa, Tsukuba, Ibaraki 305-8569, Japan

E-mail: qingshuo.wei@aist.go.jp

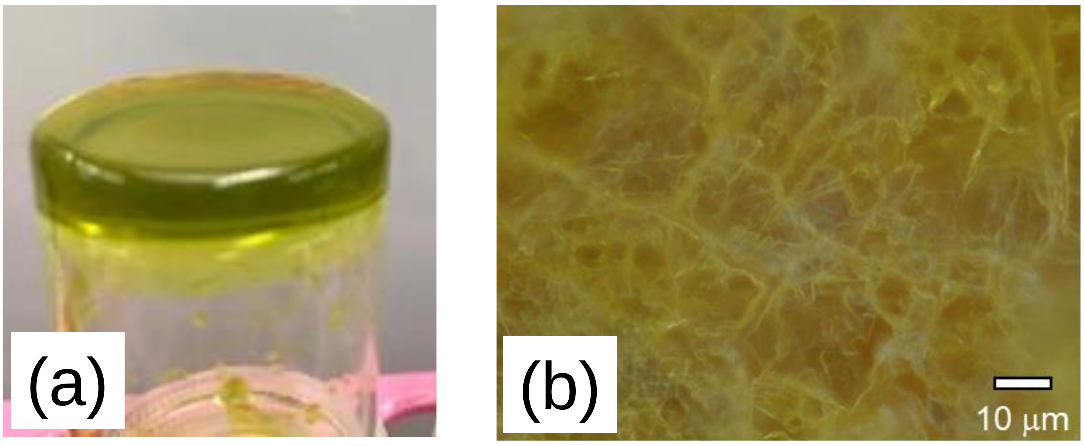

Figure S1. (a) The Photograph of the gel prepared using 1 wt% agar with the ferricyanide/ferrocyanide solution. (b) Micrograph of the gel after freeze-drying.

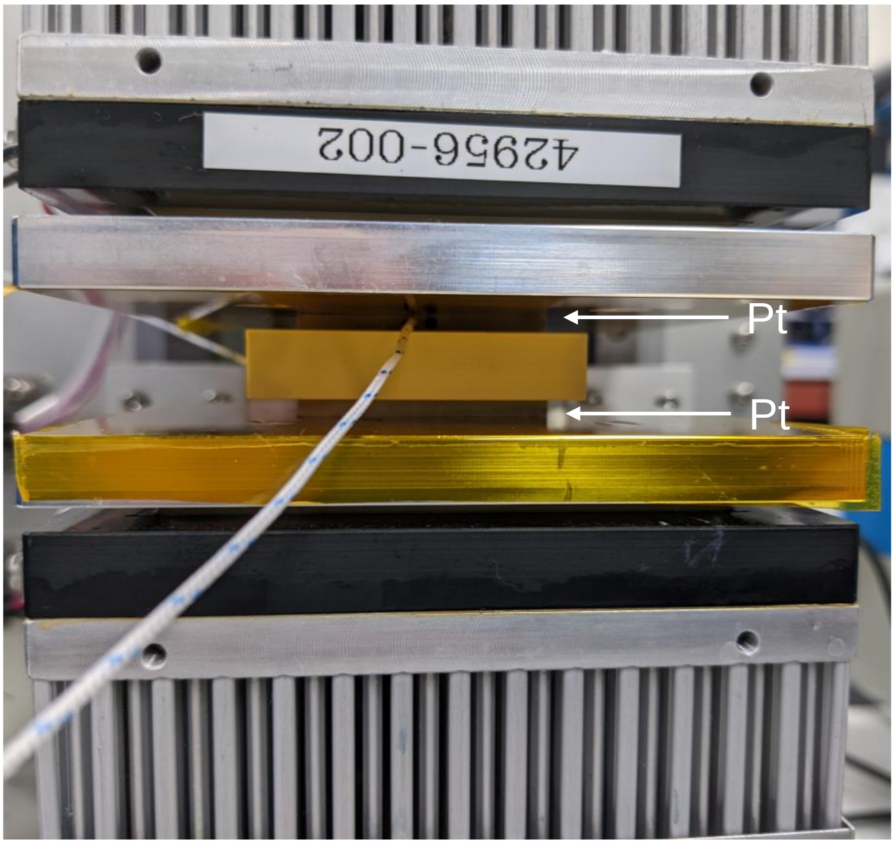

Figure S2. The Photograph of the homemade setup for thermopower measurement; the small hole at the side of the Pt electrode for the thermocouples.

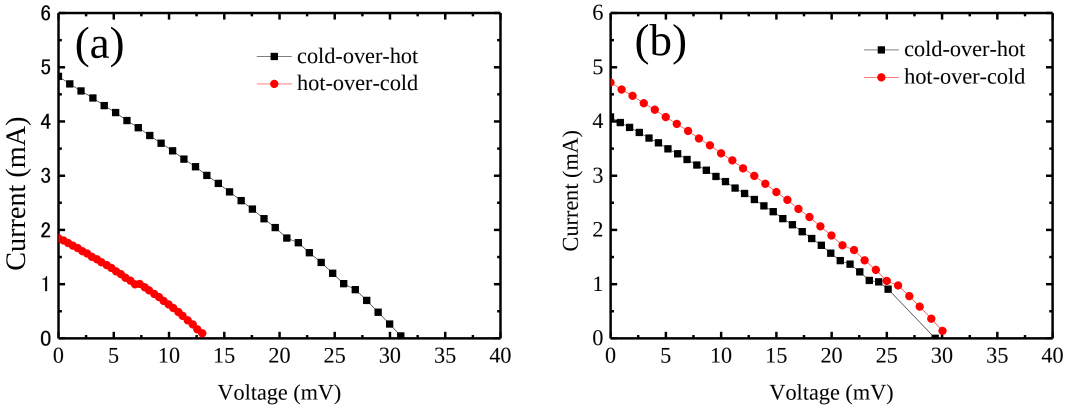

Figure S3. (a) the voltage *VS* current of thermocells with 0.4 M $[Fe(CN)_6{}^{4-/3-}]$/1 M GdmCl solution as electrolyte, and (b) the agar gel of 0.4 M $[Fe(CN)_6{}^{4-/3-}]$/1 M GdmCl as electrolyte.

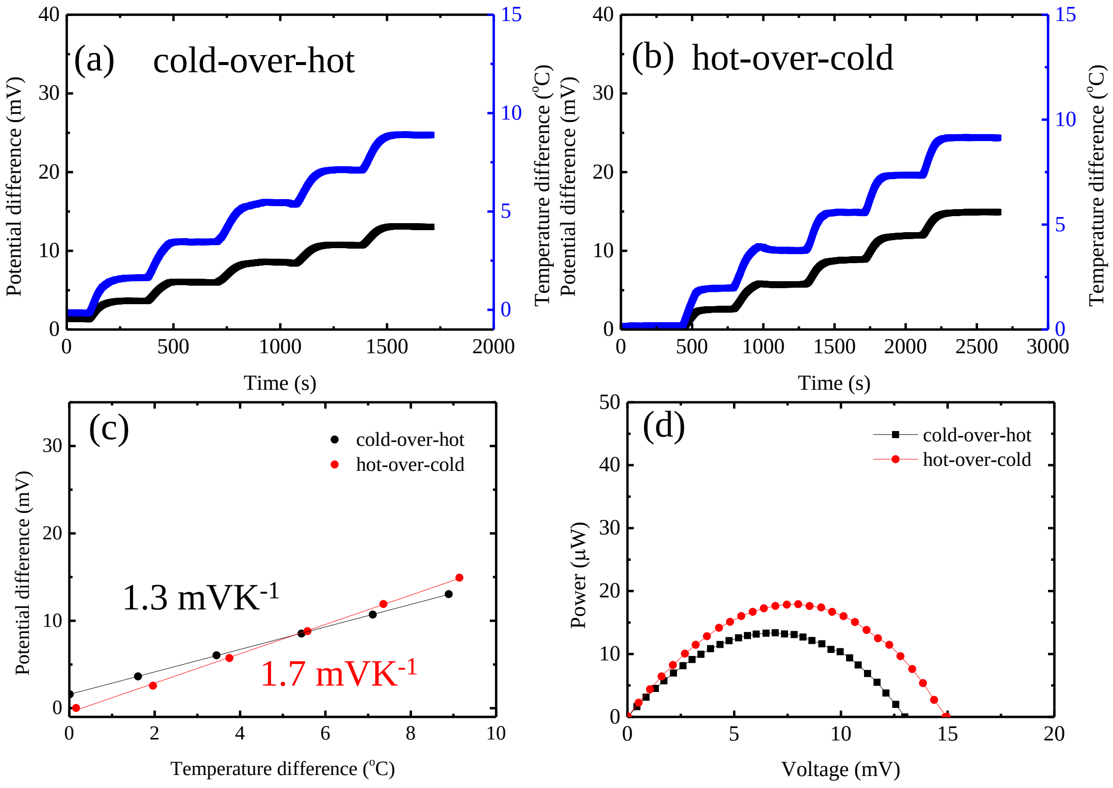

Figure S4. Temporary change in potential difference measured by Pt electrodes with the imposed temperature difference between the electrodes for (a) gel of the ferricyanide/ferrocyanide electrolyte, where the temperature of the top electrode (cold) was controlled at 25°C (cold-over-hot electrode arrangement), and (b) gel of the ferricyanide/ferrocyanide electrolyte, where the temperature of the bottom electrode (cold) was controlled at 25°C (hot-over-cold electrode arrangement). (c) Plot of the open-circuit voltage as a function of the electrode temperature difference for gels of ferricyanide/ferrocyanide with cold-over-hot (black dot) and hot-over-cold (red dot) electrode arrangements. (d) Power outputs of the devices using gels of ferricyanide/ferrocyanide characterized by a cold-over-hot (black dot) and of hot-over-cold (red dot) electrode arrangements, when the temperature of the hot side was 35°C and that of the cold side was 25°C.

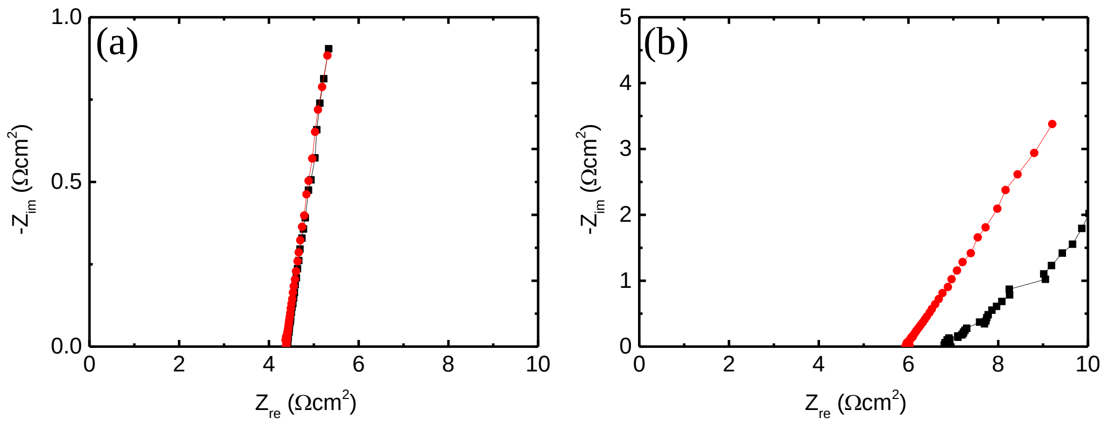

Figure S5. Nyquist plots of devices using (a) a solution of ferricyanide/ferrocyanide electrolyte (black dot) and the gel of the ferricyanide/ferrocyanide electrolyte (red dot); (b) a solution of ferricyanide/ferrocyanide/guanidinium electrolyte (black dot) and the gel of the ferricyanide/ferrocyanide/guanidinium electrolyte (red dot).

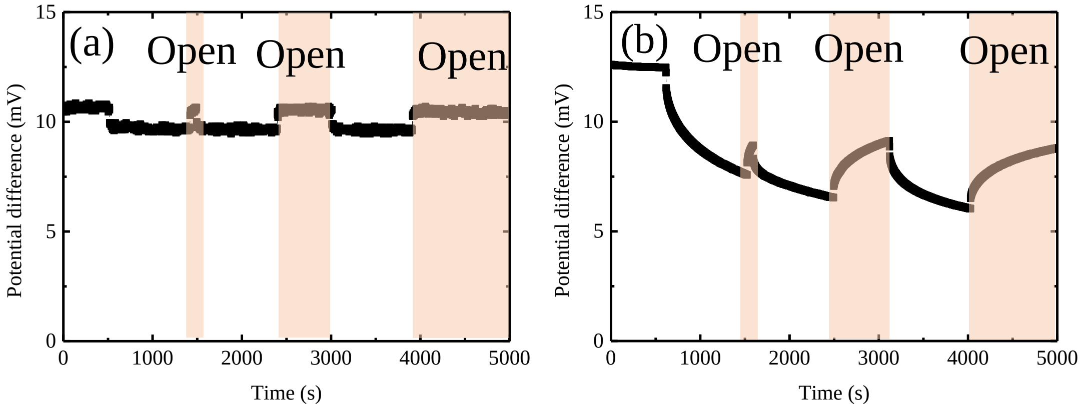

Figure S6. Temporary change in voltage during cell connection and disconnection from the external loading (40 Ω) for devices based on (a) the solution of ferricyanide/ferrocyanide and (b) the gel of ferricyanide/ferrocyanide. In both cases, the cold electrode was placed above the hot electrode.

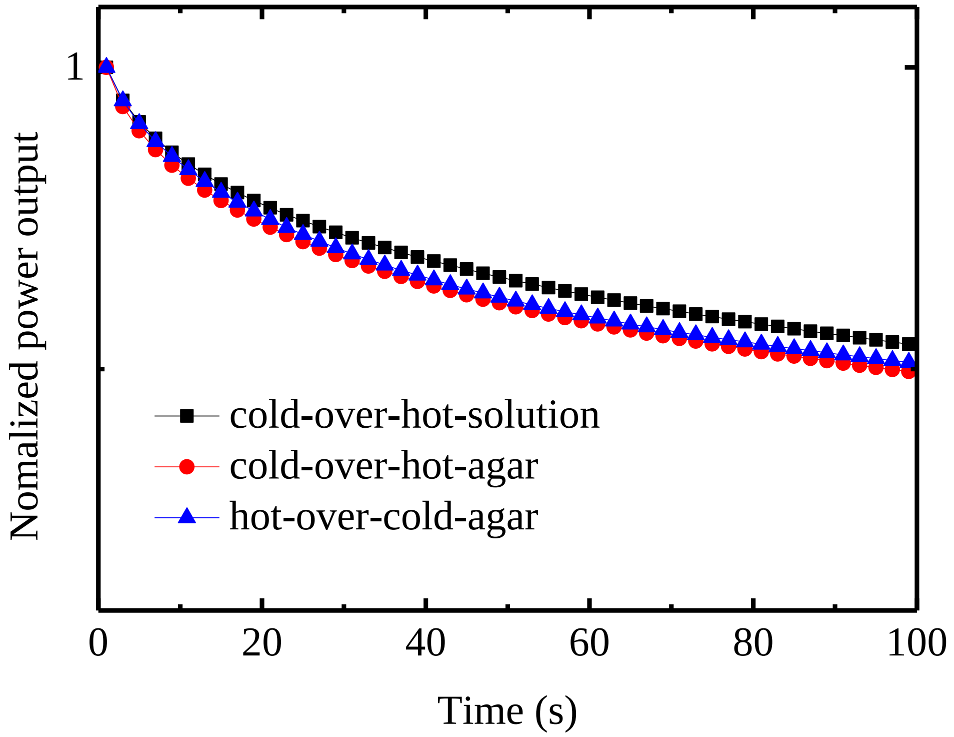

Figure S7. Plots of the power output of cells comprising ferricyanide/ferrocyanide/guanidinium electrolyte solution with external loading (40 Ω) and gel of the ferricyanide/ferrocyanide/guanidinium electrolyte as a function of the working time, whereby the cold electrode was above the hot electrode (cold-over-hot electrode arrangement) or *vice versa* (hot-over-cold electrode arrangement).
